# Extra Supplement—The Nursing Mirror

**Published:** 1889-05-11

**Authors:** 


### The Nursing Mirror.

All Contributions for this Supplement should be addressed "The Nursing Mirror," care of the Editor, 139-140, Salisbury Court, Fleet Street, London, E.C.

### En Passant.

A BAZAAR BY NURSES.—The nurses of Grimsby Hospital are to be congratulated on having originated and managed a bazaar in the new wing of their hospital. The wing was beautifully decorated for the occasion, and the stalls, which contained the usual fancy materials and useful articles, were taken charge of by the matron (Miss Montford) and the nurses. Miss A. Stephenson took charge of the bran-tub, and Miss Allen the shoe. The Countess of Yarborough opened the bazaar and accepted from the nurses a very beautiful bouquet. The proceedings all went off with great *éclat*, and a substantial sum was received for the charity.

NURSING IN AMERICA.—There is a sanatorium at Battle Creek, Mich., where a staff of eighty trained nurses are kept, who do not yet fulfil all the work which awaits them. Indeed, it is said there is work for a hundred more nurses, both men and women. Connected with the sanatorium is also a school of housewifery, where cooking, housekeeping, and all womanly duties are taught. The nurse in America is a more independent woman than she is here. As a rule, she leaves her school as soon as she is trained, and sets up for herself. Since the civil war ceased nursing has not been fashionable in America. Too many ladies then gathered an experience of the true nature of nursing to care to play about hospital wards in time of peace.

CLAPHAM MATERNITY CHARITY.—A small home has been opened at 74, Jeffreys-road, Clapham, which district nurses and district visitors may be glad to know about. It is under the medical charge of Dr. Annie McCall and her students, and married women are received at a minimum charge of 5s. per week. Other women can be received under certain restrictions, and if some responsible person will undertake such charges as the medical superintendent may deem proper. There is also a private ward. The home fills a gap in the list of existing charities, and is worthy of support. Subscriptions will be welcome. Those nurses or visitors who have difficult cases they know not where to send should apply to this home for further particulars.

HALIFAX INFIRMARY.—The nurses of the Halifax Infirmary are eagerly following the discussion on the proposed new building, for they have to suffer from the present overcrowding; their quarters are quite unsuitable for hard-working women. Mr. Whiteley Ward at a late meeting reminded his hearers that nurses nowadays were a superior class to what they were formerly. Nurses "in charge" were educated persons, and the recognised conditions of accommodation for nurses existing at other institutions of the country ought to exist at the Halifax Infirmary. In his opinion, they must go in for a new infirmary, or else they must throw upon the infirmary authorities the duty of refusing to admit so many cases. Archdeacon Brooke also said that he had visited the infirmary recently, and was struck with the noble wards, all filled, and with the signs all around of the skill and care with which the patients were tended; but he must say he was surprised that the people of Halifax should be content for a single day to let the housing of the nurses and staff be such as they were. He hoped that the money would be expended in making the new building a model one internally, and that they would care less for rivalling other towns in architectural beauties and exterior ornamentation. With two such good friends to speak in their favour, the nurses may be sure of better accommodation very shortly.

REST FOR NEWCASTLE NURSES.—At the quarterly court of the governors of Newcastle Royal Infirmary, Dr. Philipson said that the nurses were very efficient and faithful to their duties; and the chairman then announced that his wife (Mrs. Stephens) and Miss Blakwell had taken two rooms at the head of Claremont-place, to which the nurses of the infirmary would be allowed to go, two at a time each evening, and stay over night. There would be no cost to the infirmary. This would give a little holiday to the nurses, who were looking forward to it with great pleasure. A couch, one or two easy-chairs, a few pictures, and other articles were required to make the rooms comfortable, and he had no doubt that this announcement would result in everything needed being at once given. Much applause followed this speech, and it is evident that the nurses at Newcastle are appreciated and cared for. We congratulate them on having made such good friends.

RECREATION FOR NURSES.—Major Mears does not find his scheme for supplying nurses with amusement is taken up warmly by the owners of boats or carriages. Just in the rush of the season it is scarcely to be expected that ladies will often be willing to lend their carriages, yet there ought to be some who would undertake to call at a hospital once a week and take any nurse who could be spared for a drive. The scheme is a small imitation of Miss Phillippa Hicks' plan of providing nurses with homes during their holidays; a plan which has worked excellently so far. Yet there are some nurses who would feel more at their ease if not entirely domiciled in a strange family, and now the holiday season is coming we would be glad to know what nurses think of the following arrangement: Suppose certain ladies residing at the seaside or in the country, should look out for lodgings near them where nurses could be taken at 5s. a week. The nurse would then have a comfortable room of her own during her two weeks' holiday, but the lady friend should undert ke to invite her to her house, procure her the means of seeing the sights of the neighbourhood, and generally undertake not to leave the nurse or nurses dull and lonely. The question is, are there many nurses without personal friends or relations to whom such a scheme would be welcome? If there are, we should be glad to hear from them.

ARMY NURSING.—The following reply to a query which appeared in the *Queen* will be interesting to many of our readers who are constantly asking for information on this subject: "For admission to the nursing staff of the military hospitals, about which 'Cass' enquires, application should be made to the Director-General of the Army Medical Department, Victoria-street, Westminster, S.W. The nursing profession is at the present time so overcrowded, that I understand the chance of a vacancy taking place at present is small. In making an application, a declaration must be signed by the candidate, stating particulars of age, parentage, education, and previous medical training. She must also produce a recommendation from some person of standing in society to the effect that she possesses the personal tact, temper, and ability qualifying her for appointment, and that she is in every way a desirable person to enter a service composed of ladies of good social position. When these and other conditions have been duly complied with, and she is fortunate enough to obtain an appointment, she will be able, as a nursing sister, to receive a salary beginning at £30 and increasing to £50. A senior nursing sister, acting as superintendent, earns an additional £20. The lady superintendents are now generally chosen from the ranks of the nursing sisters, to whom there is therefore some hope afforded of bettering their position; the pay of the ladies who fill these posts averages from £150 to £200 per annum."

### Nursing in the Soudan.

*(Continued from page xvii.)*

Part V.—On Duty Again.

The very morning the acting superintendent reported the arrival of her party in London to the proper authority, she was directed, that having accomplished the duty of nursing the invalids' home, she and the sisters were "to turn round and go back again," to take up the work originally intended—namely, the charge of the nursing department in the Victoria Hospital at Suez. We had become sufficiently military not to be astonished at the unexpected, or to question any order given. However, we received permission for a week's holiday at home, which was eventually lengthened to more than a fortnight, during which we rested and were thankful.

A pleasant little episode during our short stay in England must be recorded, because it testifies to the warm interest always evinced by our Royal Family in the case of the sick and suffering. Their Royal Highnesses the Prince and Princess of Wales, who had visited the hospital ship "Ganges" before it left the docks (enquiring into every detail of the preparations for the sick), on our return in the "Iberia" honoured us by the request that the acting superintendent should attend at Marlborough House with one of the sisters. Their reception by Her Royal Highness the Princess of Wales was most gracious and kindly. Many questions were asked about individual cases, and the Princess entered warmly into our experiences, in which she showed very great interest, mentioning some of the sick officers by name, and desiring to know of their condition.

The authorities very kindly allowed our party to return to Egypt overland, *via* Brindisi, thus sparing us the repetition of a tedious and disagreeable voyage. We accordingly left London by the limited mail at 8 p.m. on the 8th May, and greatly enjoyed travelling through France and Italy. At 9 a.m. on Sunday we reached Bologna, and for the first time since leaving Calais changed carriages on to the Italian line. Such a glorious day! the blue waters of the Adriatic on our left sparkled in the sunlight; and with the lovely views of Italian scenery on our right, the journey was enjoyable enough. On Monday we reached Brindisi, and there changed to the s.s. "Mongolia" for the three days' steam to Alexandria. The weather fortunately was magnificent. The following afternoon our captain brought his Bible on deck, and as we steamed past the historic islands, Candia, Claudia, etc., read to us the account St. Paul gives in the 27th chapter of the Acts of the Apostles, pointing out the only spot where St. Paul could possibly have landed. On the morning of the 14th we reached Alexandria, only to find that the express (if anything could be express on Egyptian lines) had started. After some difficulty about our tickets, extra payment enabled us to travel by the only other through train that day.

Every mile of the journey in that old Biblical country was fraught with an intense interest, and one seemed suddenly transported centuries back. There were men with their wives and families tilling the land, the ox treading out the corn, the two women grinding at the mill, the ass, upon which were seated a mother and child, led by the men, just as if we were actually eye witnesses of the flight into Egypt. All round us were Old-World sights and sounds as of patriarchal times. Now and again we were rudely brought back into the present, as we had pointed out to us the battle-field of Tel-el-Kebir, and in close proximity the cemetery, where the headstones and mounds reminded us of the last resting-place of so many of our countrymen. The cemetery is well cared for; a good wall surrounds it, and the paths and graves are neatly kept. At 7 p.m. we found ourselves in the station at Suez. We looked out to make enquiries, but were disappointed to find no one to meet us.

After endless waiting, shunting, going on a little, coming back, stopping, and the like, it was found to be quite dark, when suddenly an Arab porter put his head in and informed us that they had brought us as near to the English hospital as the line would permit. This meant being put out bag and baggage on the desert sand, half a mile from our quarters, surrounded by a curious crowd of jabbering natives. After some minutes of hopeless gesticulations they caught a word or two of English known to them—"hospital," "soldiers," etc.—while some of them implied that they could take us to the hospital. Therefore, leaving two sisters to guard our baggage, we groped our way in the dark, following our guides, and at length reached the guard-house, and eventually the hospital. Not having been expected, owing to some error in the orders from headquarters, the sisters in charge had not vacated the hospital, and, consequently, there was not room for us all. For two of our party quarters were found; the others, not so fortunate, set off to the hotel at Suez, late as it was. At that moment, the interpreter and an orderly happened to make their appearance on donkeys; these men and donkeys were requisitioned, and mounted on the latter and led by the men, we made our way in the dark to Suez.

The native saddles are in no way conducive to comfort or safety used as side saddles, and thankful were we that the dark hid from view our efforts to keep ourselves from falling off. At 10.30 p.m. we at last found ourselves comfortably housed at the hotel. For ten days this pleasant hotel was our home, whilst we were waiting for arrangements to be made for the sisters in present charge of the hospital to be told off to other work. During this time of enforced inaction we obtained leave to pay a visit to Cairo, and accordingly one morning at 9 a.m. took train, and after some eight hours' journey found ourselves on the list of visitors at Shepheard's Hotel. The majority of our fellow-sojourners in the hotel were military and naval officers with their wives. In the evening after dinner the custom seemed to be, when the band played in the public gardens, for everyone to go there and enjoy the cool evening air, returning about 11 p.m. to the hotel; and we did not make ourselves exceptions to this pleasant rule. Next morning we paid a visit to the very kind and courteous officer who is head of the Medical Department of the War Office in Cairo; from him we learned that our work in the Victoria Hospital was very shortly to commence. A visit to the Pyramids, a climb to the top of one, and a scramble into the interior of another, gave us some idea of the marvellous nature of these stupendous relics of the past—3,000 years old.

Cairo is certainly a beautiful city. The most interesting parts of it are the bazaars and streets of the old town, with their wonderful mosques and temples. The heat was intense, and the glare from the white buildings of the new town very unpleasant. May is not the best month to choose for a visit to this delightful city of the East, but with us the time of year was not a question of choice. On the following Saturday we returned to Suez, and found to our great satisfaction that we were to occupy our quarters permanently at the hospital on Sunday, May 24. At last we were to have charge of the nursing of the Royal Victoria Hospital, eleven and a half weeks since we first started from home, with the object of taking up this special work.

*(To be continued.)*

### National Pension Fund for Nurses.

Hail the First Thousand!

At the meeting of the council of this fund held on the 2nd inst., Mr. Burdett in the chair, the gratifying announcement was made that 828 proposals for pensions had been received and that so many institutions had already become affiliated, that the proposals they represented would more than suffice to complete the thousand required to establish the fund permanently on the basis of its constitution. Every week additional institutions were becoming affiliated, whilst the permanent establishment of the fund will cause many who have held back to join at once. The first thousand nurses represent one in every ten of the whole body of women engaged in sick nursing, a fact of the first importance, as it shows how sound a spirit of thrift and independence animates the leaders of this section of women workers. The most popular principle of affiliation with the hospitals is one which provides for a separate trust fund for the nurses of each institution into which the authorities pay half the premiums of every nurse on their staff who joins the fund. The authorities of the nursing Sisters of St. John the Divine, having many women of good age in their employ, have generously made arrangements by affiliating themselves to the fund, which will provide a pension for every member of their staff on obtaining a certain age. The Mildmay Park authorities have adopted a similar plan, and both guarantee that all the nurses employed by them will be helped according to their circumstances. Guy's Hospital has adopted the half-premium method, whilst the authorities at Bristol and Leamington, and the Seamen's

Hospital, Greenwich, will give a proportion of each nurse's premium, varying from one-half to one-fifth of the required amount. It was reported to the council that the funds invested amounted to upwards of £36,500, whilst 693 nurses had paid their contributions amounting to £13,000, of which £700 had been received since the last meeting. The whole nursing profession has to thank these thousand nurses whose courage has practically procured for their comrades the benefit of £20,000. Never again can it be said of women that they are lacking in business knowledge or the power to combine, for here are a thousand women who, in face of much opposition, have distinctly seen the advantages of a business-like scheme, and have come forward and claimed the same. We can only say that these women are an honour to their sex.

### Our Friends Abroad.

THE following letter, just received from a nurse at Philadelphia, will be interesting to all our readers:

"I have often wished to send you a contribution, but never found time; now I am going to tell you something that took place here on Easter Day that, I think, will interest many of your readers. To-day being the Festival of the Resurrection, it was considered by the friends of the late Miss Alice Fisher a suitable occasion to hold a little memorial service at her grave. So at 4 p.m. all her old nurses (the new ones remained on duty), headed by the two chief nurses, and attended by a few of her especial friends among the doctors and their wives, walked to Woodlands Cemetery. It was a glorious day, and the chaplain's snowy surplice and the nurses' new uniforms looked very picturesque between the green grass and budding trees. Arrived at the grave, each head nurse put down the pot of tall Easter lily she carried, and the others their cut flowers, so it was a perfect altar of blossoms, round which we stood while the chaplain read appropriate prayers and collects. We sang several hymns—'The strife is o'er,' 'The King of Love my Shepherd is,' and finally that martial kind of hymn and tune for saints, commencing 'For all Thy saints in glory.' The large Winchester cross of lilies and roses, subscribed for by the nurses, was placed at the head of the grave.

"General and Mrs. Hamley and little daughter Alice are visiting in this neighbourhood, and we hope to have the pleasure of their company at our Easter hop, Tuesday night. We have a new piano in our sitting-room, and it will then be christened. Some of the chiefs, as well as the resident M.D.'s, are to grace the festival, not to mention ice-cream, without which no rightly constituted American can properly jubilate."

This pleasant picture from America makes us hope some other of our readers in far lands will send us accounts of their ways and work. To our friend in Philadelphia we send our hearty thanks for her kind letter.

### Everybody's Opinion.

[*Correspondence on all subjects is invited. No communications can be entertained if the name and address of the correspondent is not given, or unless one side of the paper only be written on.*]

### NURSING AT NICE.

MISS WOODWARD, Holland Institution, Montée de Cimiez, writes: The month of May having arrived, in which nurses who wish to join this institution during the next winter season should make application, may I ask your kind help in reminding such nurses of the method of applying and the very varied qualifications desirable, most of which were made known by your paper in March last year? First, only copies of certificates of training and of testimonials should be sent, and on *thin* paper. Postage 2½d. each half-ounce. But the original of a certificate of health must be added, given lately by a doctor, after examination with the stethoscope, etc.; a general statement of health being insufficient. All particulars must be added as to age, height, weight, date of last vaccination, and knowledge of foreign languages, and the address of a referee in a responsible position. A good general education is expected in the nurses employed, many of them being daughters of clergymen and professional men, and an ignorance of French is such a drawback that all should be prepared to take lessons on arrival here, if not able to *speak* the language fairly well beforehand. Then applicants should have had at least three years' experience, occupied either in training and responsibility in a good general hospital, or in training there, and in private nursing. A good knowledge of sick cookery is also of first importance. An interview with the directors in London in the middle of summer is necessary before coming to a decision. The date on which the nurse could conveniently manage this should be stated *clearly*, as well as her information on all the foregoing points, to save time and trouble later. Nurses are engaged for from eight to three months, between October 1 and June 1; in each case according to agreement. Salary £3 a month, laundress and travelling expenses in addition, and outdoor and indoor uniform.

### TORBAY HOSPITAL.

AN IN-PATIENT at Torbay writes: Although this hospital stands prominent among our voluntary institutions, it is a surprising fact that it is regarded by a very large class of people with a certain amount of suspicion, and is not unfrequently the subject of much unwarrantable abuse and condemnation. The Torbay Hospital and Provident Dispensary (Torquay, Devon) is, I am sorry to say, no exception. It appears to be the impression that it is a place of general discomfort, neglect, and want, and that its patients are "doctored" with extreme indifference and an utter disregard to their feelings. Now, as I am an in-patient of the above hospital, I am in a position (and other patients wish to join me) to refute this most emphatically. On the contrary, every care and attention is bestowed, both by doctor and nurse, the food is all that could be desired, and the whole conduct of the institution, with its unmistakeable *homely* comforts, speaks plainly that it is carried on, not only as a sense of duty, but as a "labour of love," the value and necessity of which is best known to those who have received its benefits. I think it most important that the true mission of an institution doing so much good should be more widely known, and would therefore ask you to give publicity to this.

### UNIFORMS AND INFECTION.

HANDCLASP writes: I am quite in the dark upon the subject of "Sister's" uniform, and under such conditions, I shall in all probability tread on someone's tender corns in my efforts to elicit information on this point. Will someone shed a ray of light on my utter perplexity? I fail to see upon what grounds the heads of hospital wards should wear dresses that will not wash, whilst the wearing of print or plain washing materials, on sanitary grounds, is rigidly enforced on all probationers and assistant nurses. I once ventured to propound my question to a matron and "sister," and received for answer the following replies: "I always give my 'sisters' dark blue serge for uniform, because it looks more dignified and marks their distinct superiority over their subordinates." This explanation, brief and concise as it was, curiously enough failed to carry conviction with it, as did also the "sister's" ingenious and confidential remark, "that really a dark dress was so becoming." Charming as no doubt these two explanations are, I still cannot reconcile them with the theoretical education I have received. We are taught that clothing carries dirt, conveys infection, and nearly all first-class authorities on nursing matters advocate the wearing of washing material for nurses' uniform, and specially insist on it for all surgical cases. Now, the probationer wears print, the assistant nurse wears print, and the "staff" wears print, but the "sister," the one who practically comes most in contact with the patients, wears dark blue, or black, week in, week out, attending septic and antiseptic cases. If it is considered so necessary on sanitary grounds for her subordinates to wear washing dresses, what reasonable excuse can she offer for wearing woollen material? The following instances have rendered my mystification complete: An ovarian operation was to take place, and the nurses to attend it were ordered not only to have everything clean on, as well as a bath, but the "sister" suggested a suspicion of carbolic might be advisable in the water; in due course the nurses, who had followed out the directions, appeared in the "theatre," and the "sister," the one who would hand the bandages, be nearest the patient, etc., was there, too, in her usual dark and "becoming" dress and very distinguishable. On another occasion, a nurse, having the care of a tracheotomy case, the operation being performed for diphtheria, was sent down on the death of the child to assist in the surgical ward, which just then was very heavy, no other precaution but a change of apron being thought necessary. Shortly after her entrance profuse hæmorrhage occurred in a recent amputation of the femor. The surgeon was sent for, the wound redressed antiseptically, and the nurse in question assisted, how far antiseptically remains to be explained. It would not be difficult to multiply such instances, but I will only give one other. I was nursing typhus—I need scarcely say such cases are filthy in the extreme, the fever attacking the lowest class of people living in the lowest parts of crowded cities. After the washing and cleansing of the patients on their entrance, it was no uncommon thing to find ourselves simply covered with pediculi, and as the sister was thoroughly good and devoted to her profession, working as hard as anybody else, she shared a like fate, but her dress could not be washed, at most it could only be "stoved"; and I for one felt thankful prints were the rule amongst nurses. I devoutly hope the day is not far distant when the starched print will be a thing of the past; but there is no need to enlarge on this point, as we are all familiar with the rustling, crackling noise of the nurse's Sunday gown.

### DISCONTENT.

SISTER MARY writes: I have been sorry to see so many letters appear in the paper from "discontented nurses." In every profession some members are hard to please, and in the nursing world, where one meets with so many grades, many to whom being "a trained nurse" means a considerable rise in the social scale, coming from the servant class of women, it is not to be wondered at. If they find themselves so hardly dealt with, why not seek their living by some other means? The institutions they condemn have paid the expense of their training, and paid their wages, etc. during the time. So that "the discontented ones" lose nothing by giving up nursing. *Good servants* are scarce, let them return to their former duties and leave the sick for those who have a love for the work, and on every sufferer can see written, "I was sick and ye visited me."

ALMSHOUSES.

J. WHITE writes: I am an old trained matron living on my savings (invested for life), but I did not live in these happy days of pension funds, and could only do my best, and that is only a pittance. If some of your good people would build a few almshouses (there is no other word that I know of to use), what a blessing they would be. All trades nearly and many professions have these almshouses, and the successful candidates are much envied. I have done my best to save, but 7s. 6d. to 8s. a week does not go far for food, lodging, and clothes, and the bit of fire an old woman needs.

PRIVATE NURSES.

UNHOLY writes: Now that the propriety of the existence of private nursing homes is in question, it would be extremely interesting if one or two nurses most competent to come to a correct conclusion would make an estimate of what fees they could command if nursing on their own account, and what their takings would be in a year, allowing for a month's leave, sick leave, and an occasional rest between hard cases. Let them imagine themselves as comfortably housed and fed when out of cases or when on duty only a portion of the 24 hours, as they are at homes, and in placing their fees they should remember that, as time goes on, the general hospital of whatever place they go to will have established homes of their own; also that every day, nay every hour, is bringing a new supply of nurses into the field.

### Appointments.

[It is requested that successful candidates will send a copy of their applications and testimonials, with date of election, to THE EDITOR, The Lodge, Porchester-square, W.]

PRINCESS ALICE HOSPITAL, EASTBOURNE.—Miss Hamilton has been appointed matron of this pleasant hospital. She trained at Barts. some seven or eight years ago, and passed an excellent examination. For the last few years Miss Hamilton has acted as sister of the Sophia Wards of the London Hospital, which post she will vacate in June to take the more responsible position at Eastbourne. Sister Edith (Miss Napper), whose departure from the Princess Alice Hospital is regretted by many, will take three months' rest before seeking another post, as the work at the hospital ever since 1883, when she was appointed matron, has been very hard. The new building is now, however, finished and furnished, and Miss Hamilton will find everything in excellent order.

SANITARY HOSPITAL, MUSWELL HILL.—Mrs. S. C. Newton, whom we gladly claim as a contributor to our pages, has been appointed matron of this infectious hospital by the Hornsey Local Board. Mrs. Newton is a trained nurse, and also a certificated masseuse and electrician. She has worked at Wolverhampton and Birmingham, and also at the West End Hospital, Welbeck-street. Her testimonials are excellent, and the board is to be congratulated on having obtained such a capable matron.

LEEK FEVER HOSPITAL.—Miss Ada Moore has been appointed matron of this hospital. She trained at the London, where she held the post of Staff-nurse Gloucester for many years. Afterwards she was appointed sister at the Cardiff Hospital, and lately she has acted as sister at Monsall Fever Hospital. Many of Miss Moore's old friends in the nursing world will rejoice to hear of her promotion.

CORK DISPENSARY.—There were six applications for the positions of midwives for the north and south dispensary districts at Cork. On a poll, Mrs. Kinihane and Mrs. Mahony were declared elected, subject to the approval of the Local Government Board.

### Lectures.

AT the Midwives' Institute and Trained Nurses' Club on May 10 a lecture will be delivered on "The Management of some Slight Deformities common in Infancy." A general committee will be held at the institute on May 17 at seven o'clock.

On May 23 Mrs. Scharlieb, M.D., will address a meeting at 18, Prince's-gardens, on the subject of the registration of midwives. Tickets can be had from Mrs. Nichol, 15, Buckingham-street, Strand.

### Examination Questions.

(The Editor will be glad of specimens of similar questions.)

(1) What precautions against bedsores should be taken in the case of a patient confined to bed?
(2) What are a nurse's duties in regard to disinfecting in fever cases?
(3) Write down apothecaries' weights and measures.
(4) How would you prevent a delirious patient from getting out of bed?
(5) How do you prepare plaster of Paris, and gum and chalk bandages?
(6) How would you prepare a private room for an operation?
(7) Explain the following terms: Anorexia, Cantharides, Caries, Cataplasm, Cluneluvium, Coma, Digitus, Electuary, Fomites, Gangrene, Linctus, Maxilla, Sinister, Syncope, Tonic, Trocar, and Viscera.
(8) What particular points should a nurse attend to in cases of Paralysis, Hernia, Ovariotomy, and Amputation?
(9) How can you tell whether bleeding is from an artery or a vein?
(10) Give the proper temperature of Cold, Tepid, Warm and Hot Baths.
(11) How do you make Beef-tea?
(12) Describe the rash in Scarlet Fever, Typhus, Typhoid, Measles, and Smallpox.

### Vacancies.

The following vacancies are announced:

**Sister.**

Hospital for Sick Children, Moor Edge, £30.

**Nurses.**

Bedford Nurses' Institute.

Chichester Infirmary (two), £30.

Eastbourne Nursing Institution.

Jersey Nurses' Institute, £25.

Ventnor Royal Hospital, £20.

Hampstead Home Hospital, £20.

Croydon Union, £22.

Bristol Infirmary (Massage).

Berry Wood Asylum, £25.

Kingston Home, Hull.

Kent Nursing Institution.

Torquay Nurses' Institution.

Guildford Hospital, £16.

Bradford Infirmary (several), £25.

Ashburton and Buckfastleigh Cottage Hospital, £25.

Nottingham and Notts. Nursing Institution, £25 to £30.

North London Nursing Iustitute.

**District Nurse.**

Hersham, Walton-on-Thames, £26. Newport, Isle of Wight, £30.

**Probationers.**

West Herts Infirmary, £10.

Burton-on-Trent Institution.

London Temperance Hospital.

Aylesbury Infirmary.

Halifax Infirmary.

### Notes and Queries.

**Queries.**

(11) *Nursing in Asylums.*—I am a trained sick nurse, and should like the charge of an asylum infirmary; to whom should I apply for such an appointment? *Boston.*

(12) *The Pension Fund.*—(*a*) If I joined the Pension Fund, Table IV., for sick pay and pension, could I receive the latter before 60 years of age, if permanently disabled, by forfeiting all claim to the bonus fund? (*b*) How many weeks is a nurse limited to, to receive sick pay? (*c*) How much would be returnable under Table IV. to a nurse's friends in case of her death? *Nurse Elizabeth.*

**Answers.**

*The Hundred Acres.*—Most hospitals, both London and provincial, take probationers of 31. You could get three months' training at a guinea a week at the London Hospital; but if you are going to take up nursing permanently you had better go to Guy's for a year. Why not go and see the matron of Croydon Hospital? *Editor.*

*Netley Hospital.*—J. M. L. should see our note this week in "En Passant" on army nursing. Probationers are not taken at Netley.

(8) *Nursing in America.*—Apply to the matron, Bellevue Hospital, New York; or the Charity Hospital, New York; or the Brooklyn Training School for Nurses, New York; or the City Hospital, Rochester, New York.

(12) *The Pension Fund.*—(*a*) In case of disablement the council would doubtless be quite willing to re-cast a nurse's policy and commence the pension at once. (*b*) Subject to certain rights there is no specified time to which sick pay is limited. (*c*) Premiums for pensions paid under the returnable system are returnable in full, but without interest, in case of death occurring before the pension is entered on.

ONE of the best disinfectants for purifying rooms, according to M. Koenig, is chloride of mercury. The room being very carefully closed, about fifty grammes of mercuric chloride are placed in a suitable vessel on a pan of burning charcoal and the apartment is left undisturbed for about four hours. The operator, on entering, should protect his mouth and nose, and throw open the windows so as to secure a current of fresh air. This process not only disinfects but also destroys all insects.

